# Seahorse Protein Hydrolysate Ameliorates Proinflammatory Mediators and Cartilage Degradation on Posttraumatic Osteoarthritis with an Obesity Rat Model

**DOI:** 10.1155/2022/4117520

**Published:** 2022-04-25

**Authors:** Sabri Sudirman, Po-Sheng Tseng, Chun-Kai Chen, David Tsou, Zwe-Ling Kong

**Affiliations:** ^1^Fisheries Product Technology, Faculty of Agriculture, Universitas Sriwijaya, Indralaya 30862, Indonesia; ^2^Department of Food Science, National Taiwan Ocean University, Keelung City 20224, Taiwan

## Abstract

Osteoarthritis (OA) is one of the age-related diseases and is highly present on the knees. Obesity and mechanical injuries as a risk factor of OA are attributed to cartilage disintegration, joint loading, and inflammation. This study is aimed at investigating the effects of seahorse protein hydrolysate (SH) on posttraumatic osteoarthritis in an obesity rat. The OA model was developed by anterior cruciate ligament transection with medial meniscectomy in a high-fat diet- (HFD-) induced obesity rat model. The male Sprague-Dawley rats were fed a HFD for 6 weeks before OA surgery. The OA rats were treated with oral gavage by 4, 8, or 20 mg/kg of body weight of SH for 6 weeks of treatment. The expressions of plasma proinflammatory factors, C-telopeptide of type II collagen, and matrix metalloproteinase- (MMP-) 3 and MMP-13 were reduced by SH treatment. Plasma superoxide dismutase and glutathione peroxidase activities were enhanced by SH. SH also relieved the pain of the knee joint and swelling as well as decreased proteoglycan loss in the knee articular cartilage caused by osteoarthritis. Based on these results, SH suppressed proinflammatory factors and attenuated cartilage degradation and pain in the OA model. Therefore, seahorse protein hydrolysate might be a potential opportunity for improving the development of osteoarthritis.

## 1. Introduction

Osteoarthritis (OA) is one of the age-related diseases and highly present on the knees, hips, and hands [1]. Symptoms of OA include degradation of articular cartilage, thickening of the subchondral bone, osteophytes, synovial inflammation, degeneration of ligaments, and menisci of the knee and hypertrophy of the joint capsule [2]. There are some risk factors associated with OA, including age, joint injury, gender, and repetitive use of the joints [3]. Obesity is also considered one of the OA risk factors due to it increasing the mechanical stress on the knee joint and adipocytes under obesity and release adipokines, such as leptin and resistin with elevated levels of proinflammatory cytokines and matrix metalloproteinases [4]. Various animal models have been used for OA experiments such as spontaneous and surgical induced OA. An anterior cruciate ligament transection with total or partial meniscectomy was used to induce a posttraumatic OA model [5]. This model mimics to naturally OA development [6].

According to the Osteoarthritis Research Society International (OARSI), the OA treatment includes physical, medical, or pharmacological and surgical treatments [7]. Among them, pharmacological treatment is the most common treatment option for pain relief and anti-inflammatory response. However, traditional medicines are limited due to failure in controlling the symptoms of OA and they cannot reverse the joint damage caused by OA. Total knee replacement surgery is considered the best treatment in advanced OA, which can effectively reduce pain and improve joint function. Unfortunately, due to the limited lifespan of artificial implants, it is not suitable for surgery in some patients [8]. Additionally, oral OA drugs may be associated with an increased risk of renal injury, gastrointestinal, and cardiovascular diseases [9]. Therefore, the emerging of novel treatments for OA management, such as through functional foods from a natural product, is a future research challenge.

Seahorse (Hippocampus kuda) is one of the members of the family Syngnathidae and has been used as a traditional Chinese medicine for many years [10, 11]. A previous in vitro study reports that peptides purified from Hippocampus kuda hydrolysate can inhibit the inflammatory response and degradation of chondrocyte extracellular matrix by blocking the nuclear factor-*κ*B and mitogen-activated protein kinase pathways [12]. A previous study also reported that this peptide inhibits collagen release on the human osteoblastic (MG-63) and chondrocytic (SW-1353) cells [13]. Seahorse hydrolysate also inhibits the intracellular reactive oxygen species level and Rac1 activation of 12-O-tetradecanoylphorbol-13-acetate- (TPA-)-induced MG-63 cells [14]. Recently, a previous study reported that Hippocampus kuda protein hydrolysate improves male reproductive dysfunction in diabetic rats [15]. However, the effect of seahorse protein hydrolysate on the OA model of obesity rats has not been reported. We hypothesized that seahorse hydrolysate has the ability to improve OA symptoms under obesity conditions. Therefore, this study was aimed at investigating the ameliorative effects of seahorse hydrolysate on anterior cruciate ligament transection and medial meniscectomy (ACLT+MMx) surgical-induced OA in a high-fat diet-induced obesity rat model.

## 2. Materials and Methods

### 2.1. Materials

Dried seahorse (Hippocampus kuda) was provided by Longwalk Marine Biotech Co., Ltd. (Kaohsiung, Taiwan). Heparin, peroxidase from horseradish, 3-(4,5-dimethylthiazol-2-yl)-2,5-diphenyltetrazolium bromide, n-(1-Naphthyl) ethylenediamine dihydrochloride, phosphoric acid, sodium nitrite, sulfanilamide, trichloroacetic acid, and Safranin-O were purchased from Sigma-Aldrich (St. Louis, MO, USA). Rat matrix metalloproteinase- (MMP-) 3, MMP-13, cyclooxygenase-2, and prostaglandin E2 ELISA kits were purchased from Elabscience Biotechnology, Inc. (Houston, Texas, USA). Rat glutathione, superoxide dismutase, total cholesterol, and triglyceride commercial kits were purchased from Randox Lab., Ltd. (Crumlin, UK). Rat CTX-II ELISA kit was purchased from Taiclone Biotechnology Corporation (Taipei, Taiwan). Rat high-density and low-density/very-low-density lipoprotein-cholesterol kits were purchased from BioVision (San Francisco, CA, USA). Rat leptin and tumor necrosis factor-alpha ELISA kits were purchased from USCN Life Science (Wuhan, China).

### 2.2. Preparation of Seahorse Hydrolysate

The seahorse hydrolysate was prepared according to Kim et al. [16]. Briefly, the dried seahorse powder was hydrolyzed with alcalase, an enzyme/substrate ratio of 1/100 (*w*/*w*), pH 7.0, and heated at 50°C for 24 hours under stirring. After that, the obtained sample was heated in a boiling water bath for 10 min to inactive the enzyme. The hydrolysate was dried by using a freeze dryer and stored at -20°C for further analysis. A previous study reported that the protein content of this hydrolysate was about 6.23 mg BSA equivalent/g dried sample with 29.04% of the degree of hydrolysis, and two major protein bands were found at 33 kDa and 63 kDa as analyzed by using a sodium dodecyl sulfate-polyacrylamide gel electrophoresis (SDS-PAGE) method [15].

### 2.3. Animal Experiment

**Figure 1:**
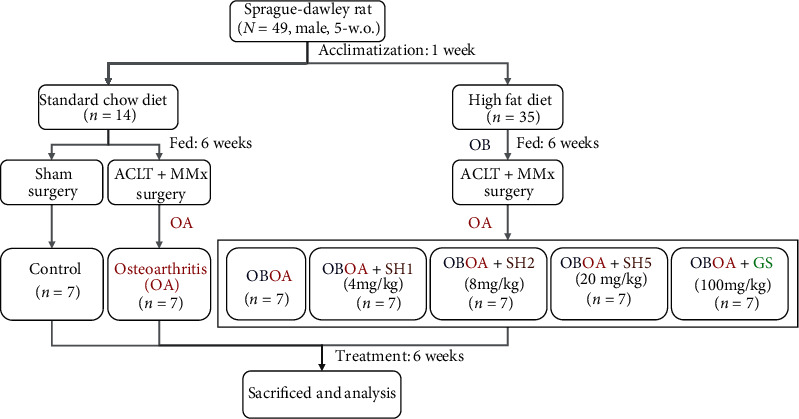
The flowchart of seahorse hydrolysate (SH) treatment on an anterior cruciate ligament transection with medial meniscectomy- (ACLT+MMx-) induced osteoarthritis (OA) in high-fat diet-induced obese (OB) rat models.

Forty-nine of 5-week-old male Sprague-Dawley rats were purchased from BioLASCO Taiwan Co., Ltd. (Yilan, Taiwan). The rats were housed individually in stainless steel cages with a 12 h dark/light cycle and fed a standard chow diet composed of 13.43% kcal from fat, 29.83% kcal from protein, and 56.74% kcal from carbohydrate (Laboratory Rodent Diet 5001, PM1 Nutrition, USA) for 1 week of acclimatization phase. The animal study was reviewed and approved by Institutional Animal Care and Use Committee (IACUC Approval No. 108019) of the National Taiwan Ocean University. Briefly, after the acclimatization phase, the rats were randomly divided into 2 groups, which were fed a standard chow diet (*n* = 14) and a high-fat diet (HFD, composed of 40% kcal from fat, 20.69% kcal from protein and 39.31% kcal from carbohydrate) (obesity group; *n* = 35) (Figure 1). After 6 weeks of feeding, the chow diet group was divided into the sham group which performed sham surgery (*n* = 7) and the OA group (*n* = 7) by an anterior cruciate ligament transection with medial meniscectomy (ACLT+MMx) surgery. On the other hand, the obesity group was further divided into 4 different groups after ACLT+MMx surgery. Seahorse protein hydrolysates (SH) were given to obesity with osteoarthritis group (OBOA group) and other groups by daily oral gavage administration. The rats were treated with 4 mg of SH per kg body weight (OBOA+SH1), 8 mg/kg (OBOA+SH2), 20 mg/kg (OBOA+SH5), and 100 mg glucosamine sulfate/kg as a positive control (OBOA+GS). The sham, OA, and OBOA groups were oral gavage daily by water. After 6 weeks of feeding, the rats were euthanized with CO_2_. Blood, organs, and operated-knee were collected for further analysis.

### 2.4. Knee Surgery

The ACLT+MMx knee surgery was performed according to Hayami et al. [17]. Briefly, Zoletil 50 (25 mg/kg body weight) was injected by intraperitoneal injection to anesthetize the rats, and the hair near the knee joint of the right hind limb was shaved and then disinfected with iodine. The skin and muscle were cut to expose the ligament, and the anterior cruciate ligament and medial meniscus were removed, whereas sham surgery was performed by only cutting the skin and muscle. After the surgery, the wound was rinsed with sterile saline. The muscle was sutured with 4-0 chromic catgut (Unik, Taiwan), and the skin was sutured with 3-0 braided silk (Unik, Taiwan). Cephalosporin antibiotic (30 mg/kg) was injected by intraperitoneal injection after surgery for 3 days to prevent postoperative infection.

### 2.5. Blood Sample Collection

The whole blood was collected by a heparinized syringe to the collection tubes and stored in a low-temperature environment. The whole blood was centrifuged (1,000× g) for 15 min at 4°C, and the supernatant (plasma) was transferred to new tubes and stored at -80°C for further analysis.

### 2.6. Plasma Biochemistry Assay

Superoxide dismutase, glutathione peroxidase, leptin, cyclooxygenase-2, prostaglandin E2, tumor necrosis factor-alpha, matrix metalloproteinase- (MMP-) 3, MMP-13, and C-terminal cross-linked telopeptide of type II collagen (CTX-II) were measured by enzyme-linked immunosorbent assay (ELISA) kits with the manufacturer's protocols, whereas nitric oxide level was measured by Griess reagent according to Sun et al. [18] and malondialdehyde level was measured by thiobarbituric acid reactive substances (TBARS) assay according to Placer et al. [19].

### 2.7. Incapacitance and Knee Width Test

Under normal circumstances, the force distribution of the hind limbs of the rats would be balanced and the balance is destroyed after ACLT+MMx surgery. The force distribution of the hind limbs of the rats was measured by a channel type rat foot support force measuring instrument with Incapacitance tester (Linton Instrumentation, Norfolk, UK). After training, the rats stand on two pieces of force sensors on a 65° inclined plate. The channel-type rat foot support force measuring instrument would measure the force of the two hind limbs, and the measurement was repeated 3 times to take the average value, whereas the width of the operated-knee joint of the rat's hind limbs was measured using electronic digital calipers and the change in joint width was observed once a week [20].

### 2.8. Knee Histopathology Staining

The knee samples of the right hind limb of the rats were fixed in 4% formaldehyde solution for at least 48 hours, and the tissue section was entrusted with Rapid Science Co., Ltd. (Taiwan). The entrusted project included decalcification of the knee joint with Decalcifier II for several days. After decalcification, the tissue was embedded in paraffin (Paraffin) and cut into 5 *μ*m thick slices. The sections were selected and stained with Safranin-O to observe the loss of proteoglycan in cartilage.

### 2.9. Statistical Analysis

The statistical analysis of the data was analyzed by Statistical Product & Service Solutions Statistics (SPSS v22.0 IBM Corp., Armonk, NY, USA). All data were expressed as the mean ± SD. The comparison of a specific group was based on a single factor difference analysis, and the statistical differences were analyzed by one-way analysis of variance (one-way ANOVA). Multiple comparisons were analyzed by Duncan's test when *p* < 0.05 indicated significant differences.

## 3. Results

### 3.1. Effects of Seahorse Hydrolysate on Body Weight, Organs, and Body Fat

The body weight of untreated osteoarthritis with obesity (OBOA) group was significantly (*p* < 0.05) higher when compared to control and osteoarthritis without obesity (OA) groups (Table 1). The weights of abdominal fat and epididymal adipose of the OBOA group were also significantly (*p* < 0.05) higher than control and OA groups. These weights were significantly (*p* < 0.05) reduced after treated with seahorse protein hydrolysate (SH) for 6 weeks, whereas a high dose of SH (OBOA+SH5) significantly (*p* < 0.05) reduced the body weight when compared to low and medium doses of SH treatment (OBOA+SH1 and OBOA+SH2, respectively).

### 3.2. Seahorse Hydrolysate Regulates Plasma Lipid Properties

The OBOA group showed high levels of plasma triglycerides (TG, 159.90 ± 25.72 mg/dL), total cholesterol (TC, 50.64 ± 16.48 mg/dL), and low-density lipoprotein-cholesterol (LDL-C, 22.94 ± 3.91 mg/dL). These levels were significantly higher (*p* < 0.05) when compared to the control groups (101.60 ± 15.06 mg/dL, 31.44 ± 2.94 mg/dL, and 14.26 ± 1.66 mg/dL, respectively). The TG, TC, and LDL-C levels were significantly (*p* < 0.05) reduced after being treated for 6 weeks by seahorse protein hydrolysate (OBOA+SH5: 44.13 ± 19.90, 31.51 ± 3.98, and 17.73 ± 3.13, respectively). Additionally, there is no effect on high-density lipoprotein-cholesterol (HDL-C).

### 3.3. Seahorse Hydrolysate Suppresses Tumor Necrosis Factor-Alpha, Leptin, Cyclooxygenase, and Prostaglandin E2 Levels

**Figure 2:**
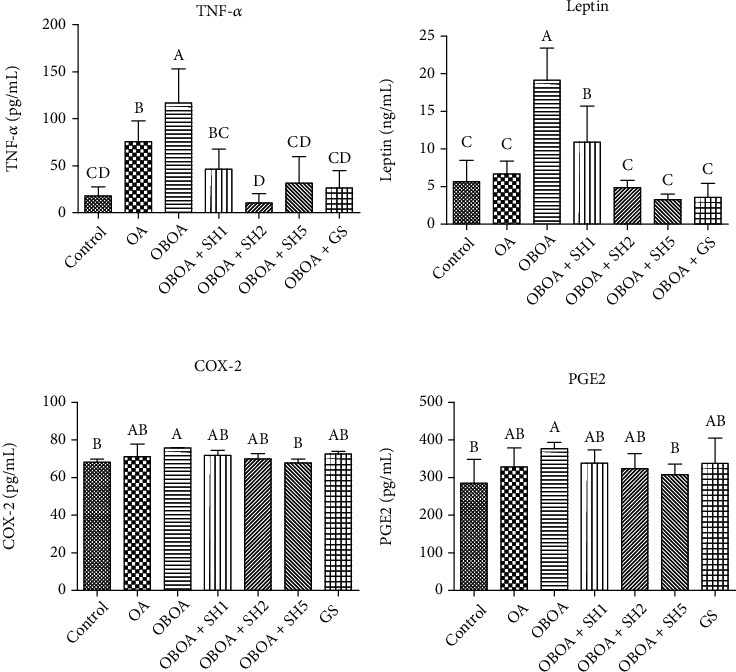
Effects of seahorse hydrolysate treatment on plasma tumor necrosis factor-alpha (TNF-*α*), leptin, cyclooxygenase (COX)-2, and prostaglandin E2 (PGE2) levels in anterior cruciate ligament transection with medial meniscectomy surgery-induced osteoarthritis in high fat diet-induced obesity rats. Data were shown as the mean ± SD (*n* = 7). The values with different letters (a–d) represent significantly different (*p* < 0.05) as analyzed by Duncan's multiple range test.

The levels of tumor necrosis factor-alpha and leptin were significantly (*p* < 0.05) higher in the OBAO group when compared to other groups (Figure 2). These levels were significantly (*p* < 0.05) suppressed by SH treatment, especially in medium and high doses of SH. These results indicated the anti-inflammatory activity of SH. The OBOA group also showed high levels of cyclooxygenase and prostaglandin E2. These values significantly (*p* < 0.05) reduced after treated with SH for 6 weeks of treatment, especially in a high dose of SH (OBOA+SH5).

### 3.4. Seahorse Hydrolysate Enhances Antioxidant Activity and Reduces Oxidative Stress Markers

**Figure 3:**
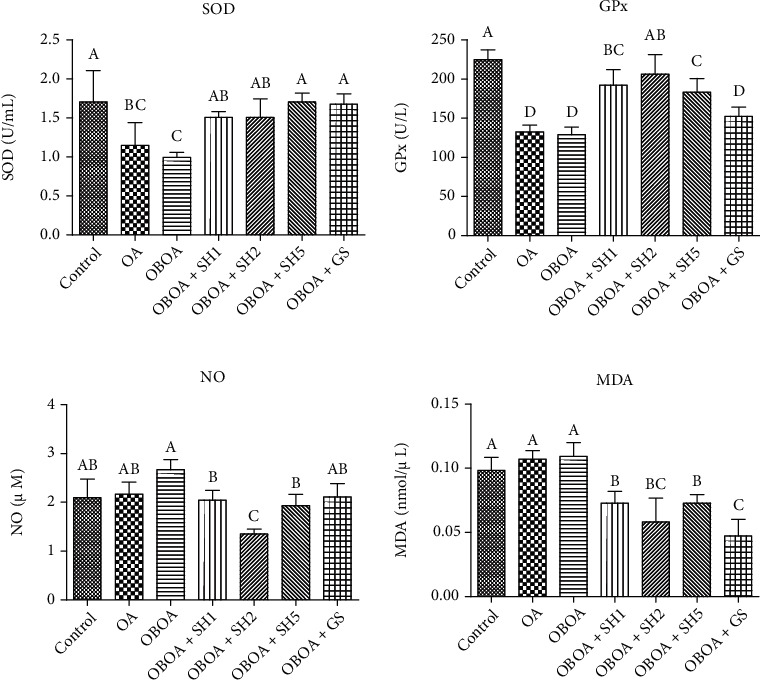
Effects of seahorse hydrolysate treatment on plasma antioxidant activity and oxidative stress markers in anterior cruciate ligament transection on medial meniscectomy surgery-induced osteoarthritis in high fat diet-induced obesity rats. Data were shown as the mean ± SD (*n* = 7). The values with different letters (a–c) represent significantly different (*p* < 0.05) as analyzed by Duncan's multiple range test. GPx: glutathione peroxidase; MDA: malondialdehyde; NO: nitric oxide; SOD: superoxide dismutase.

There are low activities of enzymatic antioxidants, such as superoxide dismutase (SOD) and glutathione peroxidase (GPx) in OA and OBOA groups (Figure 3). The SOD and GPx activities were significantly (*p* < 0.05) enhanced after treated by SH for 6 weeks of treatment. Additionally, the plasma nitric oxide and malondialdehyde levels were significantly (*p* < 0.05) lower than the OA and OBOA groups after SH treatment. The results showed that SH may have the effect of improving oxidative stress in this model.

### 3.5. Seahorse Hydrolysate Reduces Knee Pain and Swelling

After the incapacitance test (Table 2), the results showed the difference in force exerted by the feet of SH-treated groups significantly lower than the OBOA group, and there are no significant differences from the control group. The trend of the knee joint width test was also consistent with the incapacitance test (Table 3). This result showed that SH reduced swelling and pain of the knee joint.

### 3.6. Effects of Seahorse Hydrolysate on Cartilage Degradation Markers

**Figure 4:**
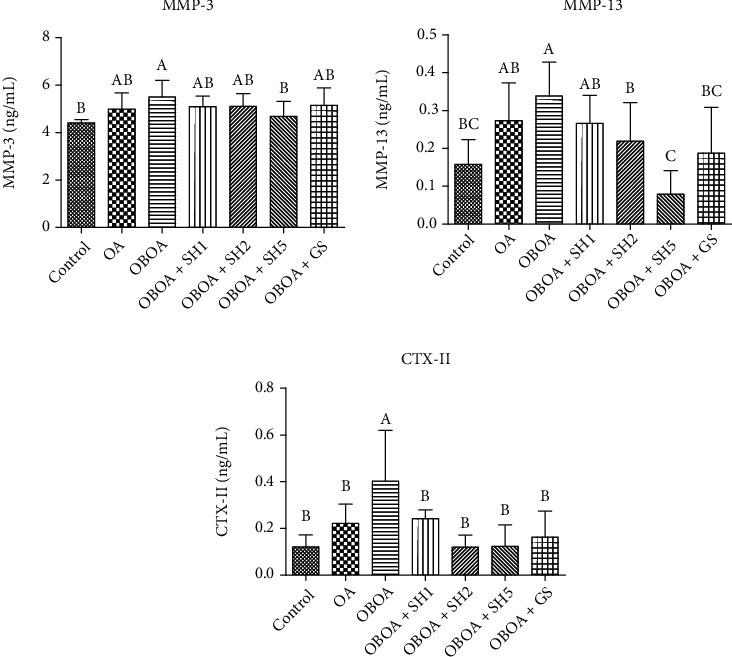
Effects of seahorse hydrolysate treatment on plasma matrix metalloproteinase- (MMP-) 3, MMP-13, and C-terminal cross-linked telopeptide of type II collagen (CTX-II) level in anterior cruciate ligament transection on medial meniscectomy surgery-induced osteoarthritis in high fat diet-induced obesity rats. Data were shown as the mean ± SD (*n* = 7). The values with different letters (a–c) represent significantly different (*p* < 0.05) as analyzed by Duncan's multiple range test.

The plasma matrix metalloproteinase- (MMP-) 3 and MMP-13 as extracellular matrix proteinases were observed an increased level in the OBAO and OA groups (Figure 4). A high-dose of SH (OBOA+SH5) treatment significantly (*p* < 0.05) reduced the MMP-3 and MMP-13 levels. Additionally, the level of C-terminal cross-linked telopeptide of type II collagen (CTX-II) as a type II collagen degradation marker was also significantly (*p* < 0.05) decreased by SH treatment. These results indicated that SH may inhibit the formation of MMPs, thereby preventing the degradation of articular cartilage. These results showed that SH improved the cartilage injury caused by osteoarthritis.

### 3.7. Seahorse Hydrolysate Improves Knee-Joint Histopathology

**Figure 5:**
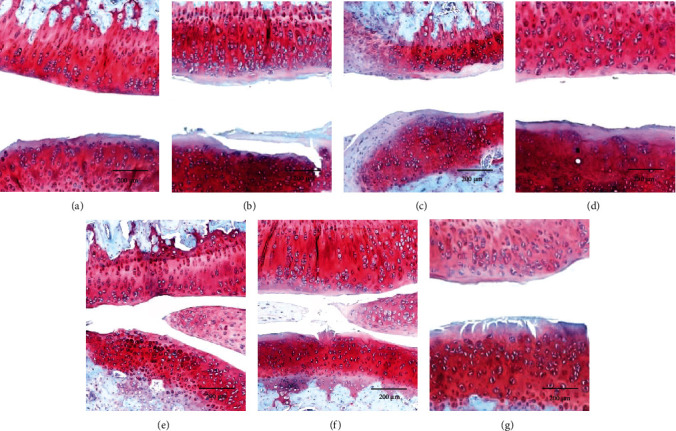
Representative of operated-knee joint cartilage with Safranin-O staining for each group after 6 weeks of treatment. Cartilage (orange to red) and nuclei (black).

According to the results of Safranin-O staining pathological sections of rat knee joints (Figure 5), the cartilage surface of the control group was intact and smooth, and proteoglycan was not lost; the cartilage surface of the OA group was uneven compared to the control group; the cartilage or proteoglycan (red region) content of the OBOA group was significantly less than OA group, and the cartilage surface was not smooth; however, the content of proteoglycan in OBOA+SH1 group was improved compared to the OBOA group, and the cartilage surface was smoother than the OA and OBOA groups; the cartilage surface and the proteoglycan contents of the OBOA+SH2, OBOA+SH5, and OBOA+GS groups were improved. Chondrocyte arrangement also was observed in normal condition and after treated with high-dose of SH and GS. However, it was disrupted in OA condition without treatment.

## 4. Discussion

In this present study, we have demonstrated oral administration of seahorse (Hippocampus kuda) protein hydrolysate (SH) to the posttraumatic osteoarthritis (OA) with an obesity (OB) rat model. The OA rat was induced by an ACLT+MMx surgery in the right knee joint. The knee surgery was performed after being fed a high-fat diet with induce obesity. As a comparison, we also developed an OA model in normal (without obesity) rats. In this case, the rats were only fed a standard chow diet. We observed an increasing body weight and adipose tissue weight of untreated osteoarthritis with obesity (OBOA) group (Table 1). Obesity has been considered a factor of OA development due to its mechanical and biochemical roles [21]. Obesity increased the loading capacity in the OA knee [22]. It also upregulated the expression of transcription factors to release some proinflammatory cytokines [23]. Daily oral supplementation of SH successfully decreased body weight and adipose tissue weight. The reduction in body weight is a nonpharmacological treatment for OA due to the decrease in the loading of knee-joint or mechanical stress of the joints [24]. Additionally, increased levels of triglycerides, total cholesterol (TC), and low-density lipoprotein-cholesterol were also observed in the OBOA group, whereas there is no effect on high-density lipoprotein-cholesterol (HDL-C). According to these data, the TC/HDL-C ratio was also elevated in the OBOA group. Oral administration of SH showed an antiobesity potential by reducing these levels.

As shown in Figure 2, the levels of tumor necrosis factor-alpha (TNF-*α*) and leptin increased in the OBOA group. Leptin is a type of adipokine, and it is mainly expressed by adipocytes in adipose tissue [25]. Leptin level is positively correlated with body fat [26]. A previous study reported that leptin is involved in OA progression as a proinflammatory mediator [27]. Leptin acts as a catabolic mediator in chondrocytes and linked to obesity and OA [28]. Other adipokines are associated with pathogenesis of inflammation, such as resistin and visfatin-1, whereas adiponectin has been known as anti-inflammatory adipokine [29]. A previous study reported that adiponectin level decreases in obese patient [30]. Additionally, adipocytes are also a source of some proinflammatory cytokines, such as TNF-*α*, interleukin- (IL-) 1*β*, and IL-6 [31]. Joint inflammation was associated with an increased level of TNF-*α* [32]. The rising level of this cytokine also has been identified in OA patients [33]. Therefore, TNF-*α* is a potential target for OA treatment [34]. In this present study, we observed that daily oral administration of SH successfully reduces TNF-*α* and leptin levels. These results indicated that SH shows anti-inflammatory properties and potential sources for OA management.

Tumor necrosis factor-*α* also plays an important role in the expression of prostaglandin E2 (PGE2) by regulating cyclooxygenase-2 (COX-2) activity [35]. In cartilage degradation, PGE2 is also considered a major catabolic mediator [36]. PGE2 is a major product of COX-2 and is related to inflammatory signs, such as swelling, redness, and pain sensation [37]. A raise in COX-2 expression was associated with cartilage degradation in OA progression [38]. A previous study also reported that elevated PGE2 is positively correlated with the severity of OA [39]. Therefore, inhibiting the activity of COX-2 is a potential treatment to reduce cartilage degradation and pain sensation in OA conditions. The OBOA group showed an increase in cyclooxygenase-2 and prostaglandin E2 levels (Figure 2).

Low levels of enzymatic antioxidants, such as superoxide dismutase (SOD) and glutathione peroxidase (GPx), were observed in the OBOA group, whereas nitric oxide (NO) and malondialdehyde (MDA) are elevated in this group (Figure 3). This condition indicated oxidative stress progression in the OBOA group. Oxidative stress is associated with various pathology processes, such as obesity, diabetes, and cardiovascular diseases [40]. Augmented generation of ROS and oxidative stress also triggers damage to the joint biochemical structure [41]. Therefore, ROS is also an important factor in OA pathophysiology. Additionally, NO is also considered to play an important role in the etiopathogenesis of OA and as a proinflammatory mediator [42]. Daily oral supplementation of SH successfully enhanced the enzymatic antioxidant and resulted in reducing oxidative stress markers. A previous also reported that seahorse hydrolysate decreased reactive oxygen species (ROS) generation [14].

Mechanical observation also supported these results as shown in Tables 2 and 3; SH administration reduced the weight-bearing difference and operated-knee swelling. A previous study reported that in many cases, OA causes pain, joint swelling, and disability [43]. In this present study, we found that daily oral administration of high-dose of SH successfully decreased COX-2 expression and resulted in a reduction of PGE2 levels. Moreover, SH treatment improved knee joint pain behavior and swelling.

High level of matrix metalloproteinases (MMPs), such as MMP-3 and MMP-13, was observed in the OBOA group (Figure 4). MMPs are a major family of extracellular matrix- (ECM-) proteinases of chondrocytes and responsible for matrix degradation of cartilage [44]. A previous study reported that MMP-3 levels increased in the synovium of OA knees and the potential to be a biomarker of OA development. It was also elevated in postmeniscectomy condition. MMP-3 is produced by chondrocytes as a response to inflammatory cytokines and under mechanical stimulation [45]. It is also produced by membrane synovium cells. MMP-3 is also known as stromelysin-1 and is responsible for the destruction of ECM in the cartilage, especially proteoglycans (PGs) [46]. MMP-13 is known as collagenase 3 and responsible for the degradation of type II, IV, and IX collagen as well as proteoglycans in cartilage [47]. Additionally, OA patients were reported to show high expression of MMP-13 [48]. An in vitro study reported that seahorse hydrolysate has ameliorative effects on MG-63 osteosarcoma cells by reducing matrix metalloproteinases (MMPs), such as MMP-1 and MMP-2 [14].

An articular cartilage is mostly composed of type II and proteoglycans, especially aggrecan [49]. Additionally, we also observed the high level of C-terminal cross-linked telopeptide of type II collagen (CTX-II). CTX-II is a byproduct during the breakdown of type II collagen and is considered a type II collagen degradation marker [50]. Therefore, inhibition of MMPs expression is an alternative treatment to reduce OA development. Oral supplementation of SH successfully inhibited the expression of MMP-3 and MMP-13 and resulted in a reduction of CTX-II levels. These biochemical data positively correlated with the histopathology analysis with Safranin-O staining (Figure 5). This figure showed that SH supplementation protected the matrix cartilage or proteoglycans loss. Based on the previous reference, Safranin-O staining showed the nuclei (black), cartilage matrix (orange to red), and cytoplasm (bluish or grey-green). Therefore, loss of cartilage or proteoglycans was indicated by the loss of red intensity [51]. Additionally, the hypertrophic condition also was observed OA condition as shown in Figure 5. During progression of OA, chondrocytes convert to hypertrophic condition and disrupted arrangement of the chondrocytes [52]. This condition also elevates inflammation progression [53].

Overall, the SH showed anti-inflammatory properties on AO with the OB rat model. However, this study is limited to the in vivo study to prove the anti-inflammation effects of SH on the OA with an OB rat model. Some further studies need to identify the molecular pathway how to the SH sample acts as an anti-inflammatory agent, such as cell-based studies. The SH sample should also be identified the compound of the amino acid.

## 5. Conclusion

Daily oral supplementation of seahorse (Hippocampus kuda) protein hydrolysate showed ameliorative effects on the posttraumatic osteoarthritis in an obesity rat model. Seahorse hydrolysate decreased some proinflammatory factors related to osteoarthritis development, such as reduction of tumor necrosis factor-alpha and leptin as well as oxidative stress. Seahorse hydrolysate also suppressed the rat's body weight. Additionally, seahorse hydrolysates also reduced catabolic mediators of cartilage degradation, such as matrix metalloproteinases and prostaglandin E2. Therefore, seahorse hydrolysate is a potential alternative for osteoarthritis management.

## Figures and Tables

**Table 1:** Effect of seahorse hydrolysate supplementation on body weight and adipose tissue weight.

Weights	Con	OA	OBOA	OBOA+SH1	OBOA +SH2	OBOA+SH5	OBOA+GS
BW (g)	474.16 ± 16.33^e^	503.98 ± 14.71^d^	661.98 ± 17.67^a^	571.60 ± 21.04^b^	574.76 ± 14.38^b^	530.25 ± 27.21^c^	495.57 ± 27.62^d,e^
% of BW							
AA	1.43 ± 0.34^c,d^	1.40 ± 0.27^d^	2.89 ± 0.69^a^	1.99 ± 0.47^b,c^	2.08 ± 0.50^b^	1.69 ± 0.40^b,c,d^	1.69 ± 0.51^b,c,d^
EA	2.09 ± 0.67^b,c^	2.03 ± 0.32^c^	4.06 ± 1.17^a^	2.91 ± 0.56^b,c^	2.97 ± 0.79^b^	2.26 ± 0.69^b,c^	2.08 ± 0.83^b,c^

Data are shown as the mean ± SD (*n* = 7). The values with different letters (a–e) represent significantly different (*p* < 0.05) as analyzed by Duncan's multiple range test. AA: abdominal adipose; EA: epididymal adipose; BW: bodyweight.

**Table 2:** Effects of seahorse hydrolysate on weight-bearing difference (*Δ* force, g) of hind limbs.

Groups	Week 10	Week 11	Week 12	Week 13
Control	6.03 ± 1.19^∗^	10.70 ± 6.19^∗^	12.88 ± 7.05^∗^	12.80 ± 5.15^∗^
OA	141.65 ± 0.49	89.97 ± 14.21^∗^	73.35 ± 10.24^∗^	56.00 ± 10.32^∗^
OBOA	140.35 ± 4.52	160.98 ± 13.53	124.95 ± 11.67	107.37 ± 13.03
OBOA+SH1	114.47 ± 6.58	55.15 ± 13.10^∗^	69.27 ± 13.52^∗^	29.93 ± 13.87^∗^
OBOA+SH2	109.18 ± 11.83	70.02 ± 14.48^∗^	40.96 ± 17.55^∗^	29.13 ± 17.03^∗^
OBOA+SH5	100.04 ± 8.89^#^	40.57 ± 17.84^∗^	29.50 ± 6.48^∗^	20.75 ± 4.64^∗^
OBOA+GS	100.66 ± 15.21^#^	65.80 ± 43.42^∗^	41.37 ± 16.12^∗^	25.34 ± 13.95^∗^

Each data point is three repetitions, which is the mean of a single 5 s reading. Data are shown as the mean ± SD (*n* = 7). Differences were considered significant at ^#^*p* < 0.01 and ^∗^*p* < 0.001 versus the OBOA group.

**Table 3:** Time-course of operated-knee joint width changes (cm) after the ACLT+MMx surgery.

Groups	Week 10	Week 11	Week 12	Week 13
Control	0.10 ± 0.00^∗^	0.13 ± 0.12^∗^	0.08 ± 0.05^∗^	0.08 ± 0.03^∗^
OA	0.20 ± 0.08^#^	0.23 ± 0.05^#^	0.23 ± 0.05^#^	0.20 ± 0.04^#^
OBOA	0.41 ± 0.03	0.34 ± 0.05	0.33 ± 0.04	0.32 ± 0.04
OBOA+SH1	0.23 ± 0.15^•^	0.11 ± 0.06^∗^	0.10 ± 0.00^∗^	0.08 ± 0.04^∗^
OBOA+SH2	0.18 ± 0.14^#^	0.13 ± 0.03^∗^	0.10 ± 0.00^∗^	0.08 ± 0.04^∗^
OBOA+SH5	0.23 ± 0.06^•^	0.05 ± 0.04^∗^	0.06 ± 0.05^∗^	0.05 ± 0.04^∗^
OBOA+GS	0.12 ± 0.04^∗^	0.07 ± 0.03^∗^	0.04 ± 0.03^∗^	0.05 ± 0.00^∗^

The width of the bilateral joint was measured every week after two weeks of surgery. Data are showed as the mean ± SD (*n* = 7). Differences were considered significant at ^•^*p* < 0.05, ^#^*p* < 0.01, and ^∗^*p* < 0.001 versus the OBOA group.

## Data Availability

The data used to support the findings of this study are included in the article.
